# Bioengineering the microanatomy of human skin

**DOI:** 10.1111/joa.12942

**Published:** 2019-02-10

**Authors:** Mathilde Roger, Nicola Fullard, Lydia Costello, Steven Bradbury, Ewa Markiewicz, Steven O'Reilly, Nicole Darling, Pamela Ritchie, Arto Määttä, Iakowos Karakesisoglou, Glyn Nelson, Thomas von Zglinicki, Teresa Dicolandrea, Robert Isfort, Charles Bascom, Stefan Przyborski

**Affiliations:** ^1^ Department of Biosciences Durham University Durham UK; ^2^ Department of Health and Life Sciences Northumbria University Newcastle UK; ^3^ Institute for Ageing and Health University of Newcastle Newcastle UK; ^4^ Mason Business Centre, Procter & Gamble Mason, Cincinnati OH USA; ^5^ Reprocell Europe Sedgefield UK

**Keywords:** barrier function, dermis, epidermis, human, methodology, reproducible, skin equivalent, tissue engineering

## Abstract

Recreating the structure of human tissues in the laboratory is valuable for fundamental research, testing interventions, and reducing the use of animals. Critical to the use of such technology is the ability to produce tissue models that accurately reproduce the microanatomy of the native tissue. Current artificial cell‐based skin systems lack thorough characterisation, are not representative of human skin, and can show variation. In this study, we have developed a novel full thickness model of human skin comprised of epidermal and dermal compartments. Using an inert porous scaffold, we created a dermal construct using human fibroblasts that secrete their own extracellular matrix proteins, which avoids the use of animal‐derived materials. The dermal construct acts as a foundation upon which epidermal keratinocytes were seeded and differentiated into a stratified keratinised epithelium. In‐depth morphological analyses of the model demonstrated very close similarities with native human skin. Extensive immunostaining and electron microscopy analysis revealed ultrastructural details such as keratohyalin granules and lamellar bodies within the *stratum granulosum*, specialised junctional complexes, and the presence of a basal lamina. These features reflect the functional characteristics and barrier properties of the skin equivalent. Robustness and reproducibility of *in vitro* models are important attributes in experimental practice, and we demonstrate the consistency of the skin construct between different users. In summary, a new model of full thickness human skin has been developed that possesses microanatomical features reminiscent of native tissue. This skin model platform will be of significant interest to scientists researching the structure and function of human skin.

## Introduction

The skin is the largest organ of the human body, accounting for approximately 16% of the total body weight. It is the primary interface between the internal and external environments which acts as a barrier to protect the body from a range of environmental stressors and maintain homeostasis by preventing unregulated water and electrolyte loss. The anatomy of human skin is well characterised, and there is a clear relationship between its structure and function. It is therefore essential to consider the structural aspects of skin when developing tissue mimetics to study skin health and disease, to ensure that the *in vitro* model accurately recapitulates the anatomical features of its native counterpart.

The skin is a complex organ with two distinct structural upper layers: the epidermis and the dermis. The epidermis is a stratified keratinised epithelium predominantly made up of keratinocytes, which is subdivided into four layers: basal, spinous, granular, and cornified. The basal layer rests upon a basement membrane at the dermoepidermal junction. It consists of mitotically active columnar cells which proliferate, migrate superficially, and sequentially differentiate to form the stratified epidermis. The basal layer is characterised by the expression of keratin 14, and the daughter keratinocytes undergo a characteristic basal‐to‐suprabasal switch from keratin 5/14 to keratin 1/10 as they move up the strata. As these cells move towards the surface and differentiate into the spinous layer, they lose their ability to divide, become larger, and establish robust intercellular connections. Keratinocytes elongate and flatten to form the granular layer, which is characterised by the presence of intracellular keratohyalin granules and lamellar bodies (Odland, 1960). Keratohyalin granules contain proteins key to the formation of the cornified envelope such as profilaggrin and loricrin (Yoneda et al. 1992). Keratinocytes are terminally differentiated into corneocytes, which make up the *stratum corneum*. During this transition, cells lose their nuclei and major organelles, lipids are released into the intercellular space and the cornified envelope replaces the cell membrane. The barrier function of the skin is mainly attributed to the *stratum corneum*, which is a 10‐ to 20‐μm‐thick layer composed of terminally differentiated, flattened corneocytes separated by layers of densely packed lipids (Menon et al. 2012).

The dermis provides crucial support to the epidermis. Unlike the epidermis, vascular and lymphatic systems pervade the dermis alongside appendages such as hair follicles, nerve endings, and secretory glands. The primary cell type in the dermis is the fibroblast. They produce extracellular matrix proteins (ECM) (e.g. collagen, fibronectin, and elastin), which contribute to the main substance of the dermis and are responsible for skin elasticity and tensile strength. The dermoepidermal junction separates the dermis and epidermis, and it facilitates the regulated exchange of substances and the polarity of the basal keratinocytes (Muroyama & Lechler, 2012). Collagen IV and integrin α6 are critical components of the basement membrane, which also contribute to the mechanical integrity of the skin.

Human skin equivalent models are important tools for academic research, clinical purposes, and industrial applications. The need for physiologically relevant models is imperative due to the recent prohibition on the use of animals for testing active compounds for cosmetics (Cosmetics regulation EC No 1223/2009). However, due to the multicellular, multi‐layered complexity of human skin, it is highly challenging to build such tissue models reproducibly and in a consistent manner. Engineered models are incrementally advancing in emulating the anatomy of skin by focusing on the co‐culture of the main cell types (keratinocytes, fibroblasts and, less frequently, melanocytes). To achieve epidermal differentiation, *in vitro* models are commonly raised to the air–liquid interface, and additives, such as growth factors and calcium, promote differentiation and stratification to recreate an *in vivo*‐like stratified keratinised epidermis (Prunieras et al. 1983; Bikle et al. 2012).

Epidermal models are most often generated on a polycarbonate membrane; however, due to the presence of keratinocytes alone, they lack the key interactions with dermal cells (Valyi‐Nagy et al. 1990). *In vivo*, the epidermis is bound tightly to the dermal component via the basement membrane, which is composed of ECM proteins secreted as a result of interactions between keratinocytes and fibroblasts (Jahoda et al. 2001). To overcome this issue, epidermal models often require the use of exogenous ECM coating to enable keratinocyte adhesion to the inert membrane.

More complex full thickness skin models include both the dermal and epidermal components. Pioneering work in the development of skin constructs initially involved seeding keratinocytes on to decellularised pig skin (Freeman et al. 1976) or human de‐epidermised dermis (Prunieras et al. 1983). More recently, the development of three‐dimensional (3D) technologies has rapidly advanced skin bioengineering within the laboratory. Full thickness models of mammalian skin have been created using hydrogel‐based technologies (usually collagen‐based) into which fibroblasts are integrated (Gangatirkar et al. 2007; Carlson et al. 2008; El Ghalbzouri et al. 2009). Although these systems provide good support for keratinocyte differentiation and stratification, there are several disadvantages, including the use of animal‐derived collagen, contraction of the collagen gel, and use of exogenous collagen matrix that can introduce batch‐to‐batch variability (El Ghalbzouri et al. 2005). Moreover, there is evidence to suggest that the particular composition and arrangement of the ECM can influence the phenotype of the dermal fibroblasts and thus their ability to support the epidermis (Maas‐Szabowski et al. 2003; Sorrell & Caplan, 2004).

An alternative strategy is to create an environment that enables the fibroblasts to generate their own ECM matrix. To achieve this, models have been generated by layering sheets of fibroblasts to mimic the dermal compartment, before the addition of keratinocytes. This approach resulted in a stratified epidermis but lacked a true 3D structure, and analysis by transmission electron microscopy showed incomplete basement membrane formation (Lee et al. 2009). More recently, we have grown primary human dermal fibroblasts in an inert polystyrene scaffold, which enables the deposition of ECM components to support overlying epithelial cells and epidermal stratification (Hill et al. 2015). However, differences in cells between donors introduced significant structural variability in the consistency and reproducibility of the models.

Although numerous approaches exist to generate *in vitro* models of human skin, a common drawback of both epidermal and full thickness systems is the complexity of the methods used to generate them. Protocols usually rely on complex in‐house media recipes that contain multiple additives such as hydrocortisone, insulin, transferrin, and epidermal growth factor (Bertolero et al. 1984). The addition of such ingredients and the use of high concentrations of serum decrease the reproducibility between laboratories and make these complex models even more difficult to replicate (Faller & Bracher, 2002; Ng & Ikeda, 2011). Moreover, the nature of cells used in skin equivalents can compromise the ability to generate skin models reproducibly. Primary cells, especially keratinocytes, are not all able to differentiate and stratify *in vitro* and can cause unpredictability (Stark et al. 1999; Eves et al. 2000). Attempts to use cell lines, such as HaCaT cells, have shown disorganised morphology (Boelsma et al. 1999; Maas‐Szabowski et al. 2003; Stark et al. 2004). Recently, Reijnders et al. successfully developed a full thickness model using TERT‐immortalised keratinocytes and fibroblasts (Reijnders et al. 2015). However, independent of the cells used, the ability to differentiate, stratify, and accurately reproduce the microanatomy of the human skin has to be confirmed. Skin models using commercially available cells, more clearly defined media, and additives would help to reduce skin structure variability significantly and enhance intra‐ and inter‐laboratory reproducibility.

Many researchers rely on commercially available systems such as EpiSkin^®^, EpiDerm™, SkinEthic^®^, and the LabCyte EPI‐MODEL 24 for epidermal models (El Ghalbzouri et al. 2008; Mathes et al. 2014), and Phenion Full Thickness, Stratatest and Epiderm‐FT for full thickness models (Schafer‐Korting et al. 2008; Ackermann et al. 2010; Rasmussen et al. 2010). A range of assays can be performed using these models, which include wound healing, skin hydration, drug delivery and phototoxicity. However, the methods used to generate these skin equivalents are not transparent, the models arrive pre‐made, and they lack the flexibility to be tailored for specific downstream applications. Furthermore, although basic characterisation of these models has been reported (Ponec et al. 2001, 2002; Ponec, 2002; Botham, 2004), it is incomplete and there is little in‐depth analysis of the microanatomy of these constructs, particularly in comparison with the structure of human skin.

In this study, we report the development of novel strategies to generate robust and reproducible models of the human epidermis and human full thickness skin that closely mimic aspects of the architecture of skin tissue. The approaches incorporate the use of batches of commercially available primary cells isolated from single donors and defined low serum media, which requires only three additives for epidermal stratification. Neonatal cells were chosen for this study due to their proliferative capacity and reduced variability, as they are derived from donors of a similar age. They enable the generation of a dermal model that does not require the addition of exogenous collagen and relies on the endogenous deposition of ECM from human dermal fibroblasts. The dermal equivalent supports the formation of a highly differentiated epidermis and the creation of a protective barrier. In‐depth analysis of the microanatomy of these models demonstrates their similarity to human skin and their use as valuable tools to study the structure and function of human skin.

## Materials and methods

### Primary cells and cell maintenance

Human neonatal epidermal keratinocytes (HEKn, Thermo Fisher Scientific, Loughborough, UK) were maintained in Keratinocyte Growth Medium composed of EpiLife^®^ medium, (Thermo Fisher Scientific), supplemented with human keratinocyte growth supplement (HKGS, Thermo Fisher Scientific) and 10 μg mL^−1^ gentamicin and 0.25 μg mL^−1^ amphotericin B (Thermo Fisher Scientific), at 37 °C in a 5% CO_2_ humidified incubator following the supplier's instructions.

Human neonatal dermal fibroblasts (HDFn, Thermo Fisher Scientific) were maintained in Dermal Fibroblast Growth Medium comprised of Medium 106 (Thermo Fisher Scientific), supplemented with low serum growth supplement (LSGS, Thermo Fisher Scientific), 10 μg mL^−1^ gentamicin, and 0.25 μg mL^−1^ amphotericin B (Thermo Fisher Scientific), at 37 °C in a 5% CO_2_ humidified incubator following the supplier's instructions.

### Skin equivalent generation

Epidermal models were generated in Millicell^®^ cell culture inserts (Merck Millipore, Beeston, UK) by coating with human collagen I diluted 1 : 100 (Thermo Fisher Scientific). HEKn were trypsinised, centrifuged, and 5 × 10^5^ cells were re‐suspended in Keratinocyte Growth Medium supplemented with 5 ng mL^−1^ keratinocyte growth factor (KGF; Thermo Fisher Scientific), 140 μm CaCl_2_ (Sigma‐Aldrich, Dorset, UK), and 50 μg mL^−1^ ascorbic acid (Sigma‐Aldrich). Cells were then seeded on the collagen‐coated insert and incubated at 37 °C in a humidified 5% CO_2_ incubator. After 2 days, the models were raised to the air–liquid interface and supplemented with 1.64 mm CaCl_2_ and maintained up to a further 28 days.

Dermal models were generated by seeding HDFn (5 × 10^5^ cells) onto inert porous polystyrene membranes (12‐well Alvetex^®^ scaffold inserts, Reprocell) and incubating at 37 °C in a 5% CO_2_ humidified incubator in Dermal Fibroblast Growth Medium supplemented with 5 ng mL^−1^ transforming growth factor (TGFβ)1 (Thermo Fisher Scientific) and 100 μg mL^−1^ ascorbic acid. Dermal equivalents were maintained for up to 35 days.

Full thickness human skin models were generated by seeding 1.3 × 10^6^ HEKn cells onto dermal equivalents in Keratinocyte Growth Medium supplemented with 10 ng mL^−1^ KGF, 140 μm CaCl_2_ and 100 μg mL^−1^ ascorbic acid, and incubating at 37 °C in a 5% CO_2_ incubator. After 48 h, the models were raised to the air–liquid interface and cultured in Keratinocyte Growth Medium supplemented with 10 ng mL^−1^ KGF, 1.64 mm CaCl_2_ and 100 μg mL^−1^ ascorbic acid and maintained up to a further 28 days.

### Human skin samples

Skin biopsies from young adult Caucasian women were collected by Procter and Gamble USA, under an IRB‐approved clinical protocol in compliance with local laws and regulations.

### Paraffin embedding

Skin equivalents and human skin samples were fixed in 10% formalin (Sigma‐Aldrich), gradually dehydrated in ethanol (30–100%) and Histoclear (Thermo Fisher Scientific), and embedded in paraffin (Thermo Fisher Scientific) using plastic moulds (CellPath, Newton, UK) to allow for transverse sectioning. Using a microtome (LEICA RM2125RT), 5‐μm sections were transferred to charged superfrost microscope slides (Thermo Fisher Scientific).

### Histological analysis

For haematoxylin & eosin (H&E) staining, sections were deparaffinised in Histoclear and gradually rehydrated in ethanol. Slides were incubated in Mayer's haematoxylin (Sigma‐Aldrich) for 5 min and rinsed in distilled water for 30 s before being incubated with alkaline alcohol for 30 s to ensure the nuclei appear blue. Samples were dehydrated, before being incubated with eosin (Sigma‐Aldrich) for 30 s, and then further dehydrated prior to mounting with DPX (Thermo Fisher Scientific) ready for microscopy using a Leica ICC50 high definition camera mounted onto a Brightfield Leica microscope.

### Immunostaining

For immunostaining, sections were deparaffinised in Histoclear and gradually rehydrated in ethanol. Antigen retrieval was achieved by incubating samples in a 95 °C water bath for 20 min with citrate buffer (Sigma‐Aldrich). Samples were then incubated and permeabilised for 1 h in blocking buffer: 20% neonatal calf serum (NCS, Sigma‐Aldrich) in 0.4% Triton X‐100 (Sigma‐Aldrich) in phosphate‐buffered saline (PBS). Primary antibodies diluted in blocking buffer (Table S1) were incubated with the samples overnight at 4 °C, followed by three washes in PBS. Samples were then incubated for 1 h at room temperature with the relevant secondary antibody (donkey anti‐rabbit Alexa Fluor^®^ 488 or 594 or donkey anti‐mouse Alexa Fluor^®^ 488 or 594, Thermo Fisher Scientific). After being washed three times in PBS sections were mounted using Vectashield/DAPI Hardset (Vector Laboratories, Peterborough, UK). The fluorescent images were captured using Zeiss 880 with Airyscan confocal microscope with zen software.

### Collagen assay

Total collagen was quantified using the QuickZyme kit (QuickZyme Biosciences, Leiden, The Netherlands) following the supplier's instructions. Briefly, snap‐frozen dermal equivalents grown for 7–35 days and standards were hydrolysed in 6 m HCl 3 mg μL^−1^ wet weight (Thermo Fisher) for 20 h at 95 °C, centrifuged for 10 min at 13 000 ***g***, and the supernatant collected. Samples were diluted in water (for a final concentration of 4 m HCl). All samples were incubated for 60 min at 60 °C with detection reagent before absorbance was read at 570 nm using a Biotek plate reader (Biotek, Swindon, UK). Total collagen values were calculated using the standard and expressed in μg mL^−1^.

### Electron microscopy

For transmission electron microscopy (TEM), samples were fixed in Karnovsky's Fixative [8% paraformaldehyde, 25% glutaraldehyde (Agar Scientific, Stansted, UK), 0.2 m cacodylate buffer (pH 7.4) (Agar Scientific) and distilled water] for 1 h at room temperature then washed three times for 5 min in 0.1 m cacodylate Buffer (pH 7.6) (Agar Scientific). Samples were further fixed in 1% osmium tetroxide (Agar Scientific) for 1 h and then washed in 0.1 m cacodylate buffer (pH 7.6), prior to sequential dehydration in 50, 70, 95, and 100% ethanol. Samples were then embedded in resin as follows: the samples were immersed in an intermediate solution consisting of a 50 : 50 mix of 100% alcohol and propylene oxide (Agar Scientific) for 15 min, moved to propylene oxide for 15 min, placed in a fresh 50 : 50 Agar 100 Epon resin : propylene oxide (Agar Scientific) mix for 15 min, and finally the samples were placed in Epon resin three times for 1 h each. The Agar 100 Epon resin was composed of 24 g Agar 100 Epon Resin, 9 g dodecenylsuccinic anhydride (DDSA), 15 g methyl nadic anhydride (MNA) and 1.4 g benzyldimethylamine (BDMA) (Agar Scientific). Then specimens were placed into rubber moulds with fresh Agar 100 Epon resin, which was left to polymerise for 24 h at 60 °C. Ultra‐thin sections were cut using a diamond knife (Agar Scientific) on a Reichert Ultracut S Ultramicrotome (Leica) and transferred to 200‐mesh copper formvar‐coated grids (Agar Scientific). Sections were then stained with 1% uranyl acetate (BDH) in 70% ethanol, washed in water, and then stained with Reynolds’ Lead Citrate (BDH) for visualisation. Ultra‐thin sections were imaged on a H7600 TEM (Hitachi).

For scanning electron microscopy (SEM), samples were critically dried (BAL‐TEC CPD 030) (Leica) and coated with 5 nm platinum, in a Cressington Coating System 328 (Cressington). Sample visualisation was performed using a S5200 scanning electron microscope (Hitachi).

### Barrier resistance assay

The integrity of the epidermal barrier was assessed by challenging the surface of the epithelium with detergent, followed by assessment of cell viability and the ability of a fluorescent dye to penetrate the tissue model. Briefly, epidermal models grown for 10 days at the air–liquid interface were transferred to 6‐well plates containing 0.9 mL of medium. To determine the concentration at which a marker chemical reduces the viability of the tissues by 50% (IC_50_) after a fixed expoure time, a range of concentrations of sodium dodecyl sulphate (SDS; 0‐4 mg mL^−1^ in water) were topically applied (70 μL) for 18 h. After exposure, samples were washed twice in PBS, dried carefully, and transferred to a 24‐well plate pre‐filled with 500 μL MTT [3‐(4,5‐dimethylthiazol‐2‐yl)‐2,5‐diphenyltetrazolium bromide 2 mg mL^−1^ in PBS, Sigma‐Aldrich] and incubated at 37 °C in a 5% CO_2_ incubator for 3 h. The MTT solution was removed, the insert was washed twice in PBS, and transferred to a clean 24‐well plate. Acidified isopropanol was added topically (300 μL) and the samples were left overnight at room temperature to allow dissolution of MTT. Absorbance was read at 570 nm using a Biotek plate reader (Swindon, UK). Relative MTT values were calculated with control cultures being set at 100%.

Barrier function of the skin models was further assessed by monitoring fluorescent dye penetration in combination with detergent treatment. Briefly, epidermal and full thickness skin models were grown for 10 days at the air–liquid interface. The surface of the models was dried, and the SDS solution (0–2% in water) was applied topically using a cloning ring (70 μL epidermal model; 150 μL full thickness model). After 1‐h incubation at 37 °C, the models were washed twice in PBS, and then 50 μL of 1 mg mL^−1^ Lucifer Yellow (Sigma‐Aldrich) was placed in the cloning ring for 20 min at 37 °C. After two PBS washes, samples were fixed and processed for paraffin embedding. Tissue sections (5 μm) were rehydrated and mounted using Vectashield/DAPI Hardset, ready for analysis using Zeiss AxioScope 40 fluorescent microscope with zen software.

## Results

### Characterisation of the epidermal skin equivalent

**Figure 1:**
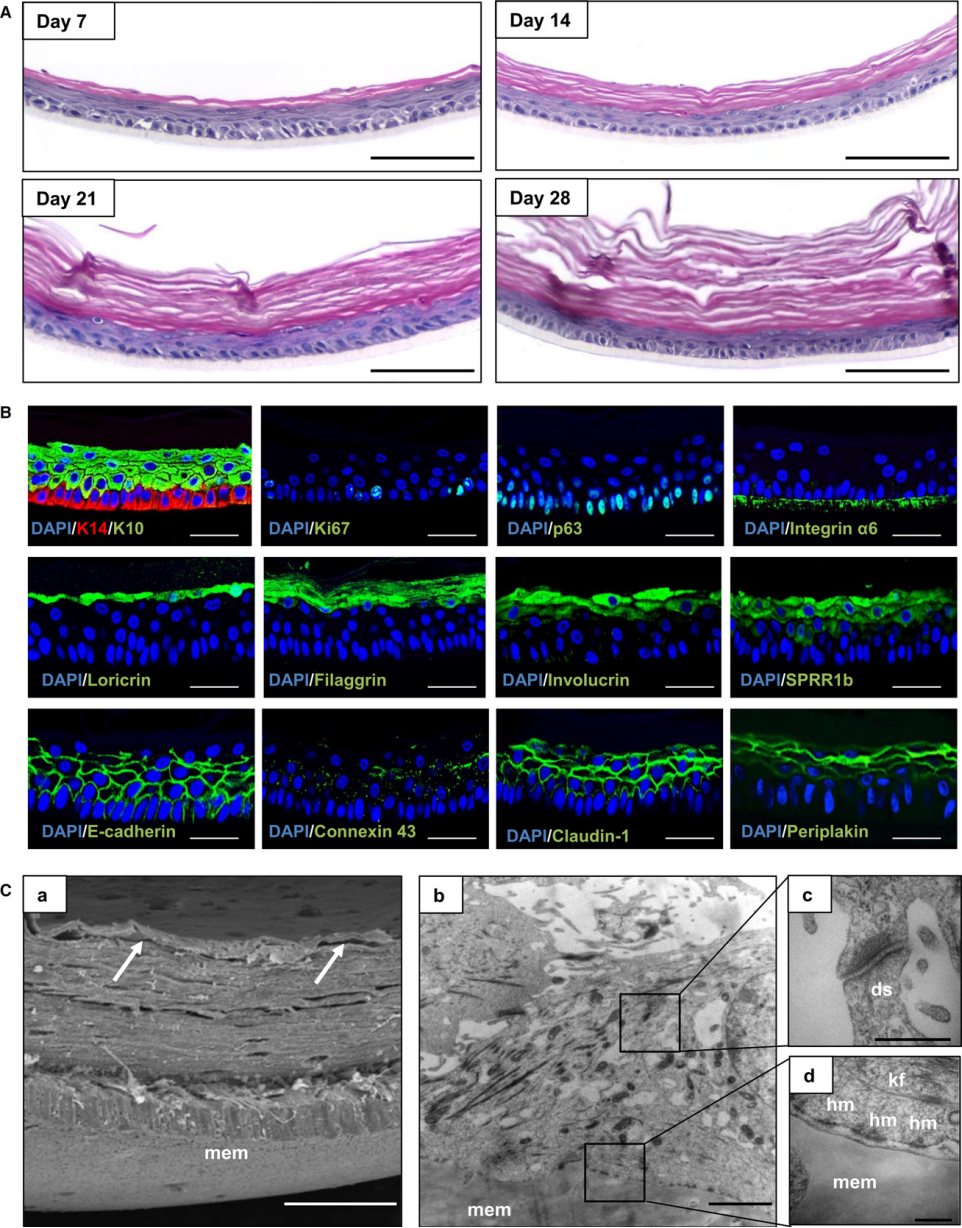
Formation and characterisation of epidermal construct. (A) Histological assessment of epidermal structure over time shown by representative H&E images of epidermal models cultured for up to 28 days at the air–liquid interface. Scale bars: 100 μm. (B) Representative immunofluorescence micrographs of the epidermal construct, cultured for 14 days at the air–liquid interface. Data show protein expression patterns for key biomarkers in the epidermis. Scale bars: 50 μm. (C) Representative SEM and TEM micrographs of the epidermal model cultured for 14 days at the air–liquid interface. (C,a) Cross‐section SEM micrographs, showing multiple cell layers with the *stratum corneum* demonstrating less adherence than the layers below (white arrows). Scale bars: 25 μm. (C,b‐d) TEM micrographs showing the Millicell® membrane (mem), hemidesmosome‐like structures (hm), desmosomes (ds), and keratin fibres (kf). (C,c) Electron‐dense desmosome between cells at higher magnification. (C,d) Electron‐dense junctional complex between cells and the underlying Millicell® membrane. Scale bars: 2 μm (C,a,b), 500 nm (C,c,d).

Histological analysis of the epidermal model over time (Fig. 1A) shows a stratified and differentiated epidermis after only 7 days at the air–liquid interface. From day 14, the organisation was similar to the epidermis of human skin. The organised columnar keratinocytes within the *stratum basale* undergo characteristic sequential differentiation to form the *stratum spinosum, stratum granulosum*, and *stratum corneum*, similar to *in vivo* skin. Terminally differentiated, keratinised corneocytes within the *stratum corneum* were also observed and stained pink by eosin. The epidermal model continues to mature for up to 21–28 days. At day 28, there was some evidence of a reduction in thickness of the lower cellular layers and an increase in cornified acellular upper layers. Figure 1B shows immunofluorescence characterisation of an epidermal model cultured for 14 days at the air–liquid interface. Similarly to mature healthy skin, keratin 14 is located exclusively within the basal keratinocytes, and the suprabasal layers express keratin 10. Basal keratinocytes also express p63, an essential transcription factor for the development of stratified epithelia, and Ki67 in the cells that are actively proliferating. The immunofluorescence data also demonstrated the differentiated nature of the epidermal model through the expression of the terminal differentiation markers loricrin, filaggrin, involucrin, and SPRR1b in the upper layers. Moreover, the epidermal models possess evidence of intercellular junctions, which are indicative of cellular communication and mechanical integrity. The presence of desmosomes, gap junctions, tight junctions, and adherens junctions within the appropriate layers was identified by periplakin, connexin 43, claudin‐1, and E‐cadherin, respectively. The gross structure of the epidermal model was analysed by SEM (Fig. 1C,a), which highlights the organised layers and the presence of flaking cells on the surface of the model, mimicking the structure of human skin. TEM data shown in Fig. 1C(b‐d) shows the ultrastructural features of the model. Electron‐dense hemidesmosome‐like junctional complexes were observed on the basolateral surface of the basal keratinocytes at the interface with the collagen‐coated membrane, and they were evenly distributed along the entire length of the membrane. Intercellular electron‐dense desmosomes were also observed between cells, and rich amounts of keratin fibres were noted in the differentiating keratinocytes.

### Maturation of ECM in the dermal construct is required to support the formation of the epidermis

**Figure 2:**
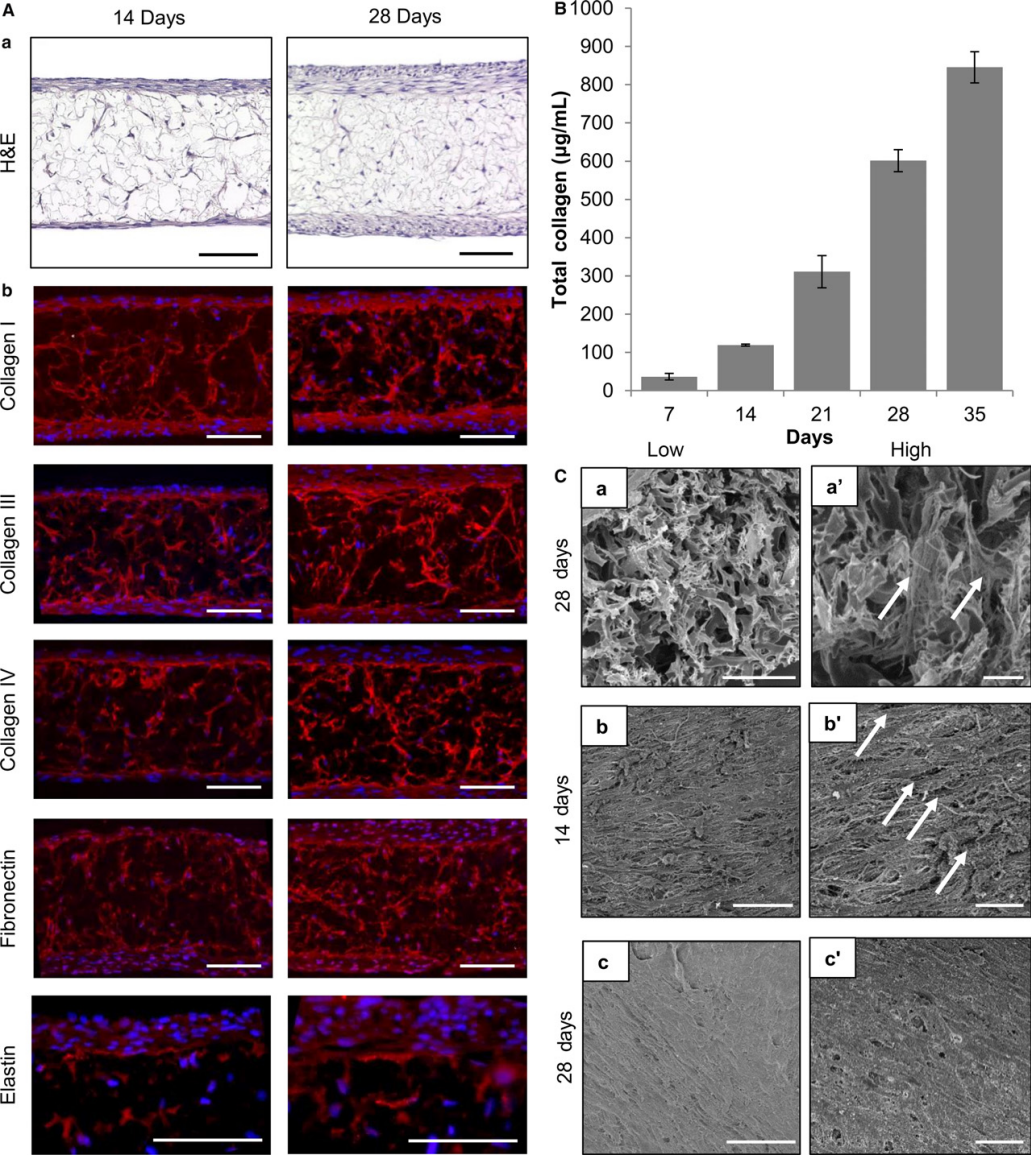
Human dermal fibroblasts grown in 3D culture deposit increasing quantities of ECM proteins over time. (A) Analysis of dermal equivalent morphology and deposition of ECM proteins. (a) Representative H&E micrographs of dermal models cultured for 14 and 28 days. Scale bars: 100 μm. (b) Representative immunofluorescence micrographs showing ECM proteins deposited within dermal models cultured for 14 and 28 days. Scale bars: 100 μm. (B) Assessment of total collagen quantification within the dermal equivalent over time. Graph showing the total amount of collagen in μg mL^−1^ as determined by a hydroxyproline quantification over 7–35 days of maturation (data represent mean ± SEM,* n* = 3). (C) Assessment of dermal model structure using SEM analysis. (C,a‐c’) Representative SEM images of dermal fibroblasts cultured in Alvetex^®^ before fixation in Karnovsky's fixative, post‐fixation in osmium tetroxide, and coating with platinum. (C,a,a’) Cross‐section of 28‐day mature dermal equivalent showing cells and ECM materials deposited within the scaffold (white arrows). (C,b–c’) SEM images of the upper surface of the dermal model show dermal fibroblasts growing on top of each other producing multiple layers at lower and higher magnification. (C,b,b’) A 14‐day dermal equivalent showing multiple large gaps between cells on the surface (white arrows). (C,c,c’) A 28‐day mature dermal equivalent where the surface is more complete with only very occasional small gaps between cells. Scale bars: 50 μm (C,a‐c), 25 μm (C,a’‐c’).

To support the formation of the epidermis and generate a full thickness model of human skin, we first investigated the ability of human dermal fibroblasts to grow within the Alvetex^®^ scaffold and deposit ECM proteins. As can be seen in Fig. 2A(a), dermal fibroblasts cultured for 14 and 28 days in the porous scaffold showed a relatively even distribution within the material. Fibroblast numbers build up inside the scaffold and also grow on both sides of the membrane, creating 3D layers of cells on the surface. After 28 days, the upper surface layer of dermal fibroblasts is approximately 30–40 μm thick. Figure 2A(b) shows a panel of ECM proteins and elastic fibre markers expressed in the dermal construct after 14 and 28 days of culture. Collagen I, III, and IV, fibronectin, and elastin were each detected within the dermal equivalent. A clear increase in staining intensity was observed over time, indicating a build‐up and deposition of endogenous ECM proteins by dermal fibroblasts within the 3D culture as the dermal construct matured.

To quantify total collagen deposition within the scaffold, an assay based on quantitative colorimetric determination of hydroxyproline residues, obtained by acid hydrolysis of collagen, was performed. This assay measures all types of collagen, which includes mature, immature, procollagen, degraded, and crosslinked collagen. Figure 2B further shows that as the dermal equivalents mature, more collagen is produced by the dermal fibroblasts within the construct over time.

Scanning electron microscopy imaging confirmed the deposition of collagen, with cross‐sections of the dermal component showing fibroblasts and extracellular collagen filaments within the scaffold (2C,a‐a’). Analysis of the surface of the dermal model showed the organisation of cells at 14 and 28 days of culture (Fig. 2C,b,c’). Gaps between dermal fibroblasts were observed at 14 days but were no longer apparent after another 14 days of growth as the culture became more dense and confluent on the surface.

**Figure 3:**
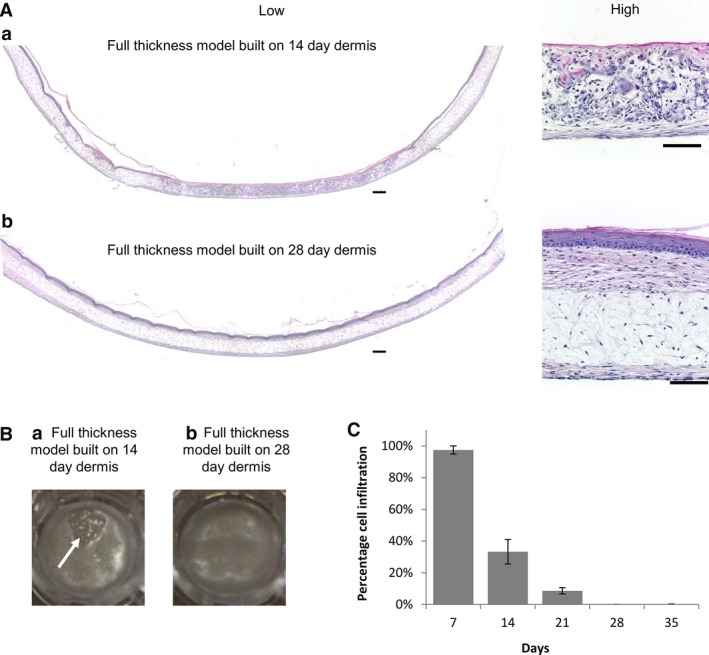
Maturation of the dermal equivalent is required to support to the growth and stratification of the epidermis in full thickness skin. (A) Differential infiltration of keratinocytes into the dermal compartment. Representative low and high magnification H&E images of full thickness models generated by culturing the dermal component for: (a) 14 days and (b) 28 days before addition of keratinocytes to the surface of the dermal model. In both cases, the models were raised to the air–liquid interface for a further 14 days to promote differentiation. Multiple low magnification images of the same cross‐section were taken and stitched together using image j software to allow the visualisation of the full length of the full thickness skin models. (A,a) There is an absence of a continuous thick epidermal layer when the dermis is cultured for 14 days due to the invasion of keratinocytes into the dermal compartment. (A,b) A continuous epidermis is formed across the entire model when the dermal compartment is matured for 28 days and no invasion is observed. Scale bars: 200 μm in full‐length cross‐section (left, low magnification) and 100 μm (right, high magnification). (B) Photographs showing the surface of full thickness skin models within the well insert. The dermal component was initially cultured for either (a) 14 days or (b) 28 days prior to the seeding of keratinocytes and culturing for a further 14 days at the air–liquid interface. A hole in the epidermis is apparent in the 14‐day dermal model (white arrow, left), whereas the epidermis is complete and uniform across the well insert in the more mature 28‐day dermal model (right). (C) Keratinocyte infiltration decreases with the maturation of the dermal equivalent. The graph shows that the percentage depth of penetration of keratinocytes into the dermal construct decreased as the dermis was allowed to mature for longer periods of time. The data were generated by capturing multiple images and assessing areas of keratinocyte infiltration using image j software to determine the depth of penetration (data represent mean ± SEM,* n* = 3).

We hypothesise that intact layers of dermal fibroblasts on top of the scaffold and ECM protein deposition within the dermal construct provide a key foundation to support the growth of keratinocytes and enable them to produce a differentiated, stratified epidermis. Figure 3A shows cross‐sections of full thickness human skin models generated using a dermal construct matured for either 14 or 28 days. Full thickness models grown on a 14‐day‐old dermal construct show areas of disorganised epidermal development where keratinocytes have invaded the dermal equivalent instead of forming an organised epidermis on the surface (Fig. 3A,a). Immature keratinocytes were identified inside the scaffold by positive expression of keratin 10 and 14 (data not shown), and a full thickness skin model failed to form. Keratinocyte infiltration was also visualised macroscopically with the naked eye (Fig. 3B). Full thickness skin models generated using a 14‐day‐old dermal construct showed visible holes in their surface. In contrast, when using 28‐day dermal models, a uniform smooth shiny surface was observed. The amount of keratinocyte penetration into the dermal construct was measured over time (Fig. 3C). Dermal equivalents cultured for only 7 days showed an almost complete penetration of keratinocytes, whereas the amount of cell infiltration reduced in correspondence with the increased culture period of the dermal equivalent, approaching zero infiltration after 28 days of maturity.

### Characterisation of the microanatomy of a full thickness model resembling the structure of human skin

**Figure 4:**
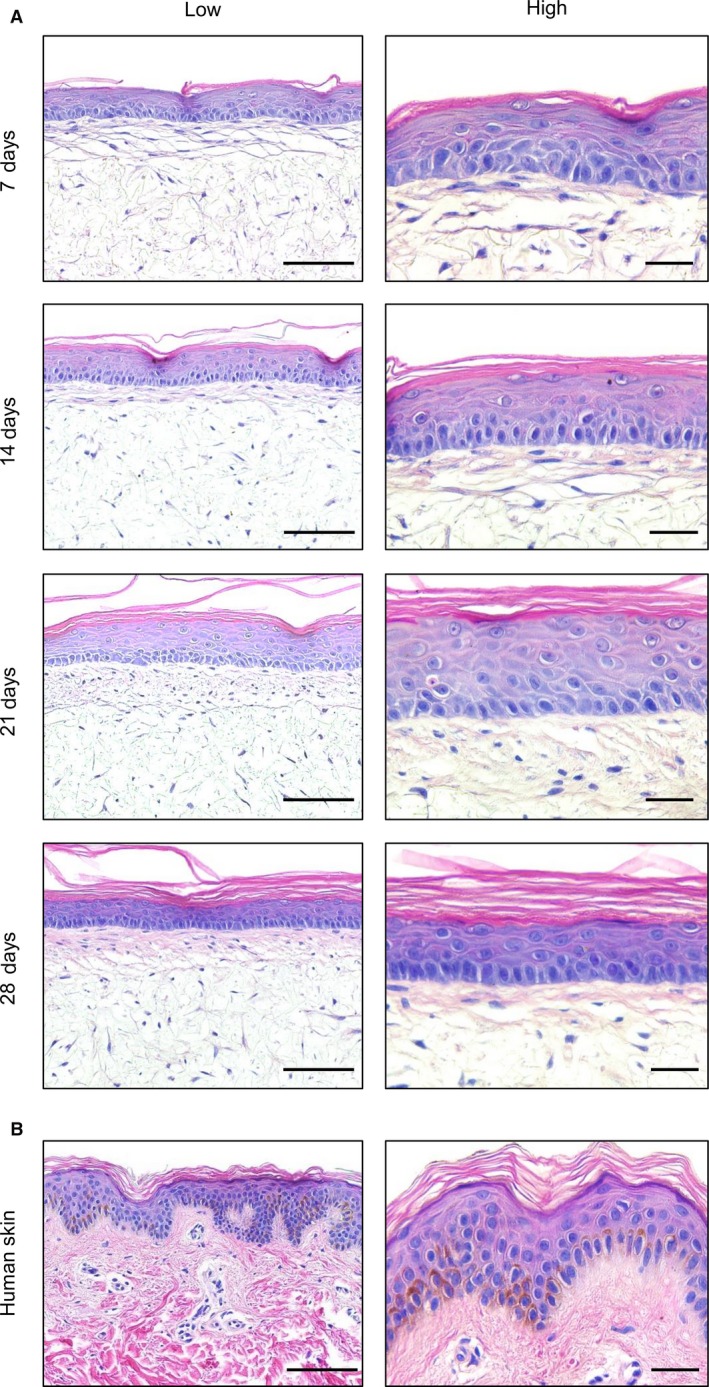
Histological analysis of the full thickness human skin model over time. (A) Representative H&E images of full thickness skin models generated using a dermis initially cultured for 28 days and then maintained for a further 7–28 days at the air–liquid interface. (B) Representative H&E images of a human skin sample from a 25‐year‐old Caucasian donor. Scale bars: 100 μm (left, low magnification) and 30 μm (right, high magnification).

The structure of the human full thickness skin model was monitored over time and compared with real skin tissue (Fig. 4). After 7 days at the air–liquid interface, the epidermis was highly organised with columnar basal keratinocytes, that undergo sequential differentiation and terminally differentiate to form acellular corneocytes, within the *stratum corneum*. From day 14, detaching layers of the *stratum corneum* were observed, indicating the maturation of the stratified epidermis. The human full thickness construct remained stable throughout the period tested, maintaining columnar basal keratinocytes and shifting keratinocyte orientation to form multiple suprabasal cell layers, which closely resemble the architecture of human epidermis after 21–28 days. For the purposes of this study, the model was maintained for 4 weeks, but in other work, the skin equivalents have been grown for several months.

**Figure 5:**
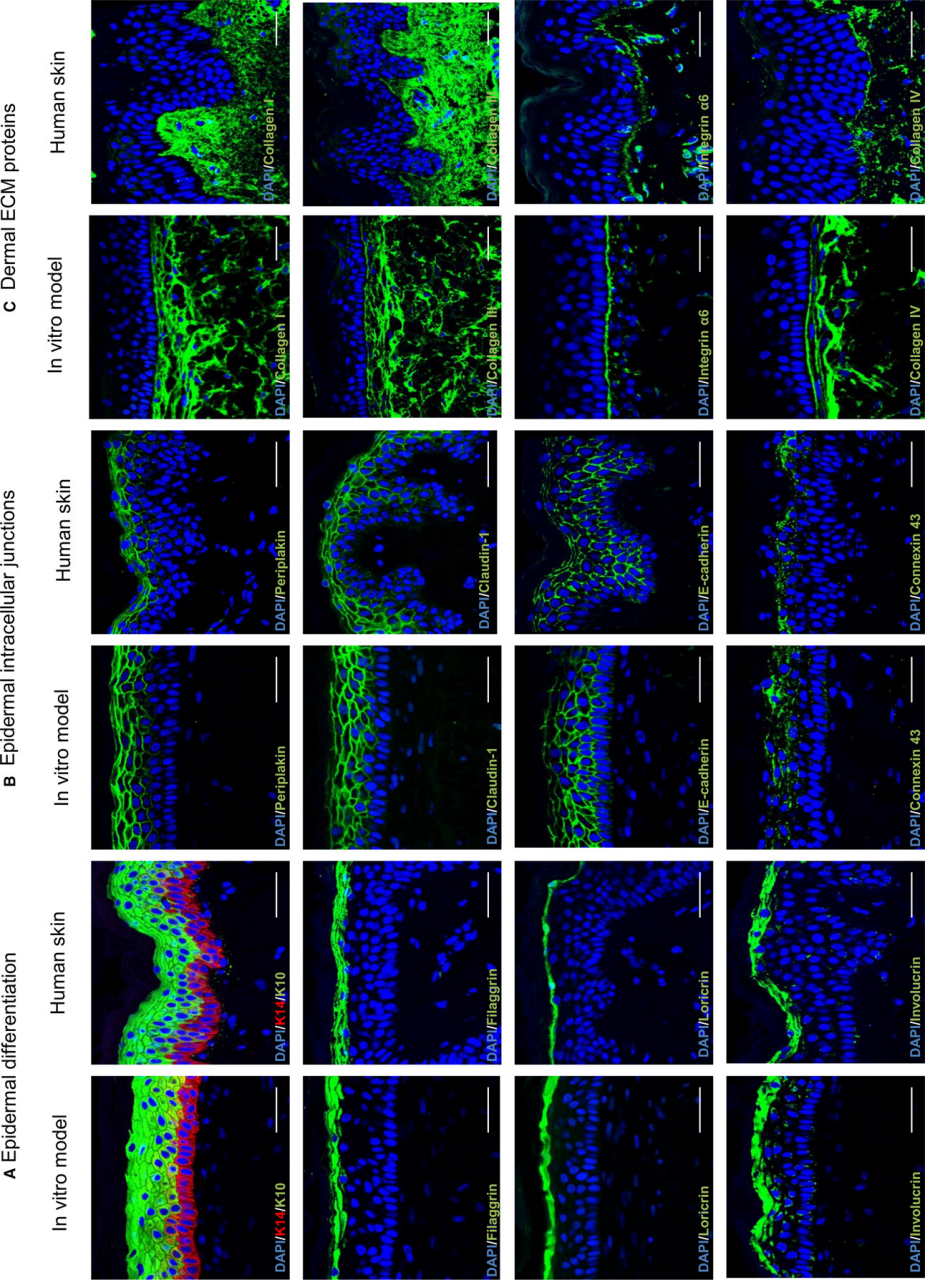
Immunofluorescence analysis of full thickness skin model compared with real human skin. The full thickness skin model was generated by culturing the dermal compartment for up to 35 days, then allowing epidermal differentiation for 14 days at the air–liquid interface, and compared with human skin from a 25‐year‐old Caucasian donor. Representative immunofluorescence images of multiple markers show very similar expression patterns for each of the proteins tested between the *in vitro* generated models and human skin. Biomarkers have been divided into three groups: (A) proteins typically associated with epidermal differentiation; (B) proteins expressed at the junctions between cells that are often associated with skin barrier; (C) extracellular matrix proteins most often found in the dermis. Scale bars: 50 μm.

Protein biomarkers representing various components of the skin structure were assessed by immunofluorescence staining in the full thickness skin equivalent and compared with human skin (Fig. 5). Markers indicative of various stages of epidermal differentiation, such as keratin 14, keratin 10, loricrin, filaggrin, and involucrin, were expressed in the model in the appropriate cell layers as found *in vivo* (Fig. 5A). In addition, four types of intercellular junctions commonly found in human skin were identified in the model (Fig. 5B): tight junctions (claudin‐1), gap junctions (connexin 43), desmosomes (periplakin), and adherens junctions (E‐cadherin). The expression of these protein markers indicates cellular communication and barrier formation within the skin equivalent. Moreover, proteins involved in the basement membrane bridging the dermis and the epidermis such as collagen IV and integrin α6 were observed.

**Figure 6:**
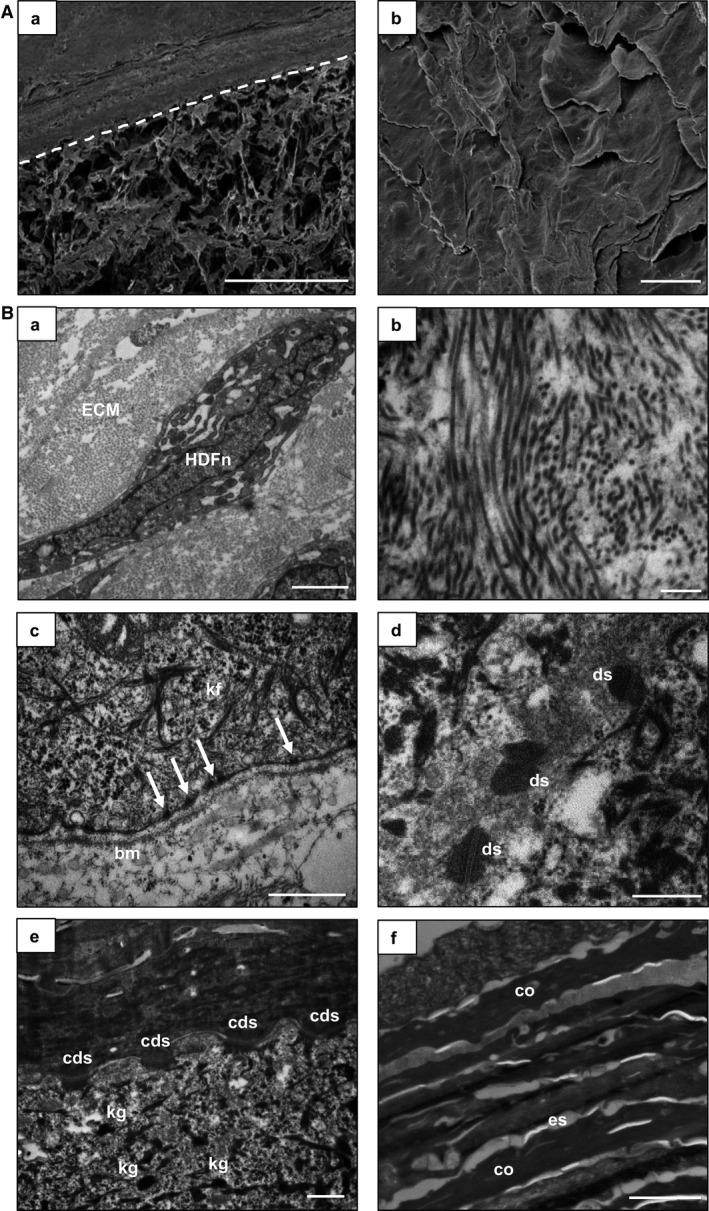
Ultrastructural characterisation of full thickness human skin model. Representative SEM (A) and TEM (B) images of the full thickness model, which includes a 28‐day established dermal compartment and the epidermis was differentiated for a further 14 days at the air–liquid interface. (A,a) SEM image of a cross‐section of the full thickness model. The white dotted line locates the upper surface of the Alvetex^®^ membrane. Scale bar:  200 μm. (A,b) SEM image of the top surface of the skin model showing corneocytes which have an overlapping, flattened appearance. Scale bar: 20 μm. (B,a) A single dermal fibroblast surrounded by ECM. (B,b) Profiles of collagen fibrils. (B,c) Interface between the dermis and epidermis showing the typical electron‐dense tri‐laminar structure of the basement membrane (bm), hemidesmosomes (white arrows), and keratin fibres (kf). (B,d) electron‐dense desmosomes (ds) between cells in the spinous layer. (B,e) Keratohyalin granules (kg) in the granulosum layer and corneodesmosomes (cds) at the interface between the cornified and granular layers. (B,f) Extracellular spaces (es) and corneocytes (co) in the *stratum corneum*. Scale bars: 2 μm (B,a,f); 500 nm (B,b‐e).

Electron microscopy and ultrastructural analyses were undertaken to visualise the architecture of the full thickness model more closely (Fig. 6). SEM analysis clearly showed the layered structure of the full thickness skin model, comprised of the dermal and epidermal components (Fig. 6A,a). Visualisation of the cornified layers by SEM confirmed the presence of flaking corneocytes (Fig. 6A,b). TEM analysis was also used to examine the ultrastructural features of the full thickness skin model (Fig. 6B), and it confirmed the presence of fibres characteristic of extracellular collagen within the dermal compartment. These ECM proteins were produced endogenously by the fibroblasts to form a dense network (Fig. 6B,a). Magnified images demonstrated that these fibres were longitudinally and transversely arranged, as in the human dermis (Fig. 6B,b). A well‐defined basement membrane was observed at the dermoepidermal junction, which was uniform and consistent across the full model, and possessed the characteristic tri‐layered pattern (Fig. 6B,c). The epidermal basal keratinocytes were tightly attached to the basement membrane via electron‐dense hemidesmosomes, which link the keratin intermediate filaments with the underlying basal lamina (Fig. 6B,c). Electron‐dense desmosomes could be observed between adjacent keratinocytes in the suprabasal layers, contributing to the mechanical integrity of the skin model (Fig. 6B,d). Corneodesmosomes were also observed at the interface between the *stratum corneum* and the *stratum granulosum* (Fig. 6B,e).

The *stratum granulosum* layers can be identified using TEM analysis by the presence of keratohyalin granules, lamellar bodies, and lipid droplets, similar to *in vivo* skin. These layers terminally differentiate to form the *stratum corneum,* which possessed a characteristic alternating electron‐dense and electron‐lucent organisation (Fig. 6B,f). Collectively, these ultrastructural features are consistent with the microanatomy of human skin.

### Reproducible generation of epidermal and full thickness human skin models

**Figure 7:**
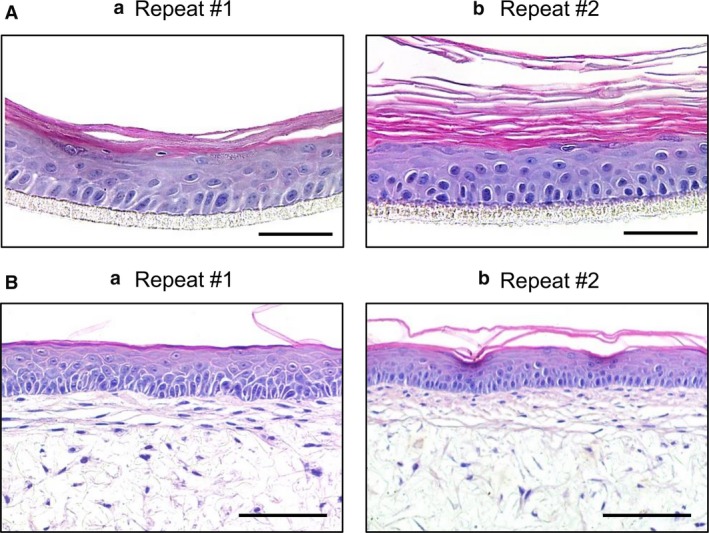
Reproducibility and robustness of the skin models. Construction of each model has been performed by independent investigators and different laboratories to demonstrate the reproducibility of the models following the methods described herein. Examples of repeat constructs for the epidermal and full thickness models are shown in (A) and (B), respectively. The epidermal models were cultured for 14 days at the air–liquid interface, and the full thickness skin models were cultured for up to 35 days for the dermal compartment and 14 days at the air–liquid interface. Scale bars: 100 μm (A); 50 μm (B).

One of the challenges of producing bioengineered *in vitro* models of human tissues is the ability to consistently reproduce what has been created and previously reported. To assess the reproducibility of the skin models, different laboratory members have been involved in generating the epidermal and full thickness skin equivalents, both in the same laboratory and in the other laboratories involved in this collaborative study. Figure 7 shows evidence of reproducibility of the epidermal (Fig. 7A) and full thickness (Fig. 7B) skin models generated by different individuals.

### Assessment of barrier function in epidermal and full thickness models

**Figure 8:**
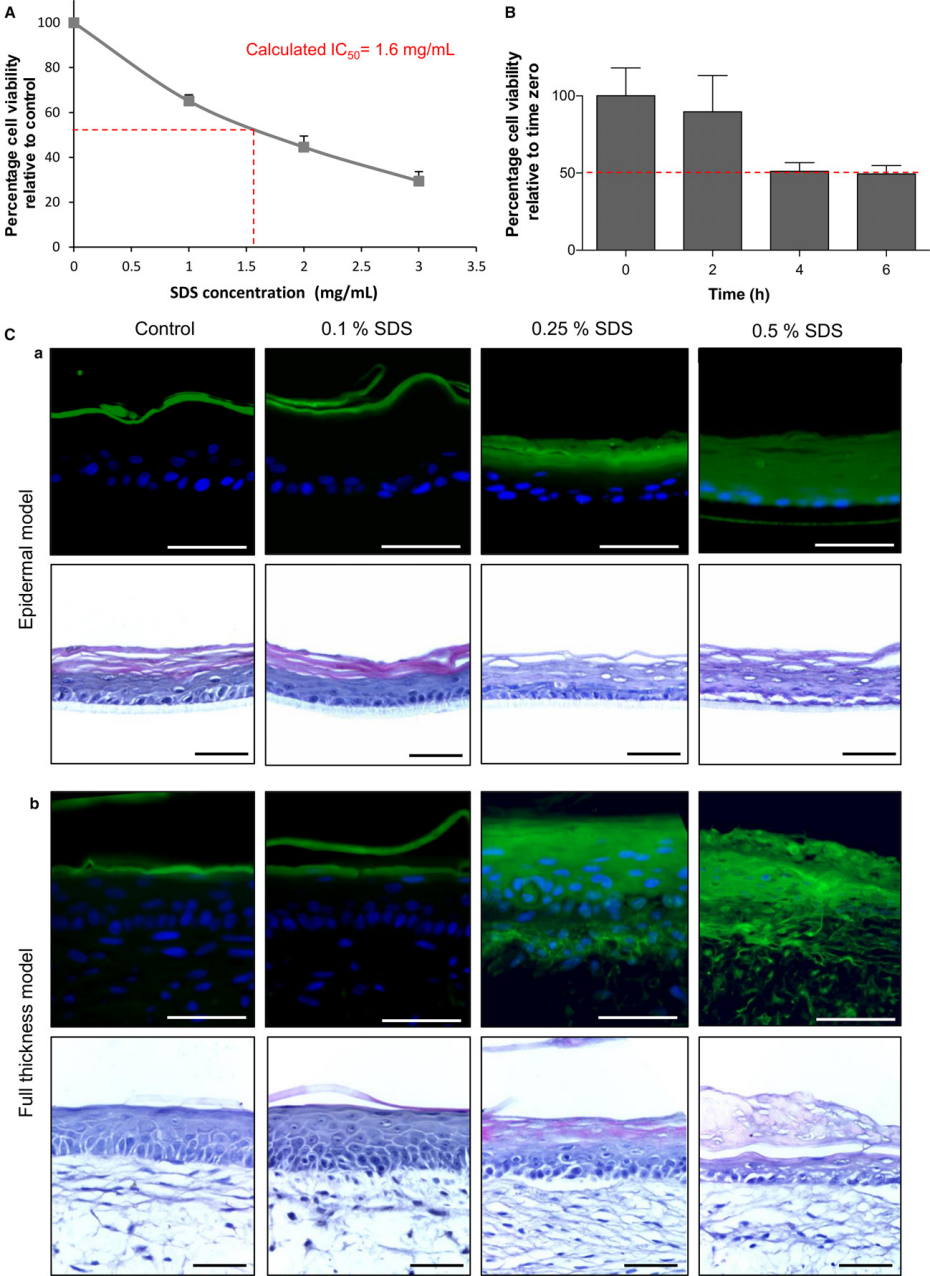
Assessment of barrier properties in epidermal and full thickness models. (A) Analysis of metabolic activity (MTT conversion) in the epidermal model cultured for 10 days at the air–liquid interface before being exposed for 18 h to a concentration range of SDS to determine an IC_50_ value. Relative MTT values are shown with vehicle control cultures being set at 100% (data represent mean ± SEM,* n* = 3). (B) Analysis of metabolic activity (MTT conversion) in the epidermal model cultured for 20 days at the air–liquid interface before being exposed to 1% Triton X‐100 for up to 6 h. Relative MTT values are shown with 0 h cultures being set at 100% (data represent mean ± SEM,* n* = 2). (C) Assessment of barrier resistance in epidermal and full thickness skin models: representative fluorescence and H&E images of epidermal (C,a) and full thickness models (C,b) cultured for 14 days at the air–liquid interface. Samples were exposed to a concentration range of SDS for 1 h, followed by the application of the green fluorescent dye lucifer yellow for 20 min to assess the penetration of dye diffusion and barrier integrity. Note the penetration of the dye at the concentration of 0.25% SDS and higher, and the damage inflicted to the surface of the culture model. Scale bars: 100 μm.

Assessment of the integrity of the barrier in the epidermal and full thickness skin models was undertaken by challenging the surface of the structure using detergents (Fig. 8). The viability of epidermal models cultured for 10 days at the air–liquid interface was assessed in response to 18‐h exposure to increasing concentrations of SDS (Fig. 8A). An IC_50_ value of 1.6 mg mL^−1^ SDS was calculated as the effective concentration whereby 50% of the cells remained viable, which is within the OECD acceptance limits for OECD 431 and 439 (Skin Corrosion and Skin Irritation tests, respectively; OECD, 2015, 2016). We also confirmed that the epidermal models cultured for 20 days at the air–liquid interface could withstand exposure to 1% Triton X‐100 for up to 6 h, with 50% of the cells remaining viable (Fig. 8B). In addition, visible disruption of barrier function was demonstrated by exposure of the epidermal and full thickness models to increasing concentrations of SDS for 1 h prior to the topical application of the lucifer yellow fluorescent dye (Fig. 8C). Lucifer yellow, an aqueous‐soluble dye, was retained in the *stratum corneum* when topically applied to untreated models or constructs topically exposed to 0.1% SDS. As the SDS concentration increased to 0.25% or above, there was evidence of lucifer yellow penetrating the *stratum corneum*. Histological examination showed that there was injury to the cells beneath the *stratum corneum* at these higher concentrations.

## Discussion

Developing strategies for the production of *in vitro* models that recreate the structure of human tissues has multiple benefits, including use in fundamental research to improve understanding of cellular mechanisms and signalling pathways, and many applications in drug screening, safety assessment, and disease modelling. The ability to accurately recapitulate the architecture of human tissues *in vitro* is critical to the success of these models. It is a well‐founded principle that structure is related to function. The closer the similarity between the structure of *in vitro* models and their native counterpart, the more useful the tissue equivalent becomes in terms of more accurately recapitulating tissue function. The structure–function relationship is a fundamental principle in anatomy and physiology that has stood the test of time. In the modern cell culture laboratory, new technologies such as 3D cell culture can be applied to enhance the behaviour of cultured cells significantly. 3D cell culture alters the microenvironment in which cells grow, influences cellular interactions, and changes their structure and function, to enable the creation of more tissue‐like structures. In this study, we have developed new strategies to accurately recreate aspects of the microanatomy of human skin. We have successfully developed a full thickness skin equivalent that possesses barrier function properties. These models are robust and reproducible as they are generated using batches of commercially available cells, off‐the‐shelf consumables and defined low serum media. In‐depth comprehensive characterisation of these tissue equivalents has demonstrated anatomical similarities with human skin.

Human skin has a complex multi‐layered structure. In healthy skin, keratinocytes lose their columnar shape and undergo cytoarchitectural changes as they differentiate, stratify, and become keratinised. In this study, this process has been accurately recapitulated *in vitro*. The skin equivalents possess distinct layers reminiscent of the four main layers of native human epidermis, the basal, spinous, granular, and cornified layers, which have been confirmed by the expression of protein biomarkers representative of the appropriate stages of epidermal differentiation. Importantly, the models are fully humanised and the full thickness model, unlike many others, does not require the addition of animal‐derived exogenous ECM proteins. Human fibroblasts within the dermal compartment produce their own endogenous ECM to support the differentiation and stratification of the overlying epidermis.

Skin equivalents are already widely used to understand biological processes, especially for cosmetic purposes or drug testing. Moreover, since the European prohibition of animal testing for cosmetic products and cosmetic ingredients (Cosmetics regulation EC No. 1223/2009), the need for physiologically relevant human skin equivalents has grown significantly. Several models have already been developed (Zhang & Michniak‐Kohn, 2012), but producing a model that provides consistency and reproducibility between laboratories has proven difficult. Variability in the production of skin models results from several factors, including the source of cells, medium composition, operator contribution, and the techniques used. In this study, we have addressed these issues to improve the consistency and reproducibility of skin equivalent tissue structure. Firstly, to minimise variation of the source of cellular material within a study, we access different lots of commercially available cells that are first batch‐tested as described herein and are readily accessible to the scientific community. Additionally, we have shown that the full thickness model can be generated using different lot combinations of fibroblasts and keratinocytes, offering greater flexibility. Secondly, there are multiple media recipes in which skin cells have been isolated and cultured, leading to a lack of consistency of practice. Historically, epidermal cell isolation has been performed using many media additives (Rheinwald & Green, 1975; Bertolero et al. 1984), and it is well known that high concentrations of serum in media can differentially affect the culture of fibroblasts (Guo et al. 2016). Accordingly, reproducibility between different laboratories becomes almost impossible (Faller & Bracher, 2002; Ng & Ikeda, 2011). To help address this issue, we used defined commercially manufactured media with the addition of three additives: KGF to promote keratinocyte proliferation and stratification (Marchese et al. 1990; Andreadis et al. 2001), ascorbic acid involved in epidermal differentiation (Savini et al. 2002), and calcium, known to promote keratinocyte proliferation under 0.1 mm and to encourage keratinocyte differentiation at higher concentrations (Hennings et al. 1980; Bikle, 2012). Thirdly, introducing hydrogels to create a full thickness skin model, primarily in the form of collagen‐based materials from different sources such as rat, bovine or human, can generate batch variability (El Ghalbzouri et al. 2005) and may introduce species‐specific effects when combining animal and human materials. We overcome this issue by not introducing any exogenous ECM components in the construction of the full thickness model and by allowing the dermal fibroblasts to create their own ECM within an inert porous scaffold. Gelatine‐based materials have been used for similar applications in skin models (Imparato et al. 2017), but the stability of this material does not lend itself to a robust and reproducible system. The Alvetex^®^ scaffold used in this study provides an inert and stable physical 3D microenvironment that enables cells to more closely recapitulate their native morphology (Maltman & Przyborski, 2010). Porous polystyrene scaffolds have previously been used successfully to develop complex multicellular tissue structures (Hill et al. 2015; Marrazzo et al. 2016). However, the use of primary cells directly from patients in our previous work demonstrated the issue of donor‐to‐donor variation (Hill et al. 2015), further highlighting the need for cells to undergo quality control prior to their use in skin models, which can be achieved through the use of commercially available cells. Collectively, these features have resulted in methods to engineer a reproducible human skin equivalent that has been successfully transferred between laboratories and accurately reproduced.

In‐depth characterisation of the morphology and ultrastructure of the skin equivalents showed very close similarities to the microanatomy of human skin. In the dermal compartment, we observed the expression of collagen I, collagen III, collagen IV, fibronectin, and elastin, and the accumulation of ECM as the dermal construct matured. We hypothesise that the increase in ECM deposition over time is critical to support the formation of the epidermis. Insufficient maturation of the dermal construct resulted in the invasion of keratinocytes into the scaffold, which correlated with lower levels of ECM accumulation during the first 2 weeks of growth. ECM components are produced endogenously by the resident dermal fibroblasts, and build up within and on the surface of the dermal construct, providing a suitable foundation for the formation of the epidermis. The dermal construct itself provides a useful stand‐alone model to study ECM deposition and remodelling in human dermis.

The human epidermis shows a characteristic differentiated and stratified tissue structure composed of the basal layer, spinous layer, granular layer, and cornified layers. In the epidermal construct, immunofluorescence analyses showed that markers of basal, suprabasal, and terminally differentiated cells were expressed in the appropriate corresponding layers, as observed in human skin (Hohl et al. 1991; Steinert & Marekov, 1995; Mischke et al. 1996; Blanpain & Fuchs, 2009). The presence of specialised junctional complexes between cells was also confirmed by immunofluorescence analysis, consistent with previous work (Boelsma et al. 2000).

Ultrastructural analyses provided a more in‐depth characterisation of the epidermis in both the epidermal and full thickness models. Epidermal layers are formed through programmed sequential differentiation, and they have well‐defined characteristics associated with their stage of differentiation. Columnar keratinocytes of the *stratum basale* are usually one cell thick and contain hemidesmosomes on their basolateral surface to anchor them to the basal lamina. As the keratinocytes differentiate into the *stratum spinosum*, they become polyhedral in shape and are connected by intercellular desmosomes, which are specialised to withstand mechanical stress. Within the *stratum granulosum,* keratinocytes elongate and flatten. The fundamental characteristics of this layer include the presence of intracellular keratohyalin granules, lamellar bodies, and lipid droplets. During terminal differentiation, keratinocytes undergo cornification into anuclear, flattened corneocytes within a concentrated intracellular lipid network (Eckhart et al. 2013). Our data confirm the presence of these complex, ultrastructural features within the appropriate layers of the epidermal constructs. Moreover, corneodesmosomes were identified at the interface between the *stratum corneum* and underlying *stratum granulosum,* which are rarely reported in the literature. Immunostaining and ultrastructural data also demonstrate the presence of desmosomal junctions and indicate the formation of additional types of specialised intercellular junctions typically found in human skin, including tight junctions, gap junctions, and adherens junctions (Vinken et al. 2006; Brandner et al. 2015). These data collectively suggest the existence of cellular cross‐talk to support tissue integrity and homeostasis programming.

A key structural feature of epithelium is the basement membrane, which forms at the dermoepidermal junction. Immunostaining for collagen IV and integrin α6 provided an indication of the presence of a basement membrane; however, detailed structural analysis confirmed the typical trilaminar structure of the basal lamina found in normal human skin (Chan, 1997). This complex structure forms *de novo* as the epidermal and dermal components come together and it was observed as a consistent, uniform layer across the entire model. Relatively few previous studies have reported this level of complexity, and there is evidence of variability along the length of other tissue models formed (Auxenfans et al. 2009; Lee et al. 2009; Reijnders et al. 2015; Tokuyama et al. 2015; Higgins et al. 2017). The basement membrane is a key component of skin anatomy (Halim et al. 2010) and functions strongly to anchor the basal keratinocytes to the underlying dermal component. The integrity of the basement membrane is a quality indicator of a successful skin equivalent (El Ghalbzouri et al. 2005).

Our morphological data demonstrate that the skin equivalents produced in this study share anatomical characteristics with native human skin. A primary function of normal healthy skin is to act as a barrier between the interior and exterior environments (Bazzoni & Dejana, 2002; Elias, 2008). We hypothesised that the skin equivalents possessed a functional barrier due to the presence of appropriate differentiation markers, junctional proteins, a keratinised *stratum corneum*, and a fully developed basement membrane. Assessment of barrier function is often performed in the topical administration of compounds as a convenient route for drug delivery (Naik et al. 2000; Huzil et al. 2011), and a series of standardised tests have been developed to evaluate barrier function. Most often, epidermal models are used to assess new compounds in comparison to known corrosive or irritant reagents following OECD international regulatory guidelines (Skin Corrosion and Skin Irritation tests; OECD, 2015, 2016). However, before using a skin model for such testing, the functionality of its barrier should be evaluated using reagents that erode barrier structure, such as SDS or Triton X‐100 (El Ghalbzouri et al. 2008; Mathes et al. 2014; OECD, 2015, 2016). The barrier of the epidermal model demonstrated a penetration profile for SDS and Triton X‐100, which is similar to accepted models. Franz‐type diffusion cells are often used to assess barrier integrity and transport across the skin using full thickness skin equivalents in absorption tests (Bertolero et al. 1984; Schafer‐Korting et al. 2008; Ackermann et al. 2010; Rasmussen et al. 2010). Using a recognised similar approach (Indra & Leid, 2011), we demonstrated barrier integrity in the full thickness model by showing resistance to penetration of the aqueous‐soluble lucifer yellow dye until challenged by detergent. Collectively, these data indicate that these skin equivalents possess functional barrier activity consistent with existing systems.

In summary, we have developed defined procedures to engineer human skin tissue *in vitro* in a reproducible and consistent manner. We have demonstrated the similarity of their microanatomy to native human skin using detailed morphological and structural analyses. We also report functional data consistent with the formation of an intact and robust barrier, which conforms to OECD regulatory guidelines. In addition, the full thickness model relies on the formation of endogenous human ECM proteins, thus avoiding the use of exogenous animal‐derived materials and improving consistency. We consider these developments to represent a significant advantage over previously reported systems, which are not as well characterised and which show variability (Ponec et al. 2001, 2002; Ponec, 2002; Botham, 2004; Netzlaff et al. 2005). We propose that our novel full thickness model of human skin is suitable as a platform for developing new applications to study human skin health and disease. This system is suitable for the introduction of higher levels of complexity including addition of other skin cell types such as melanocytes and immune cells to simulate inflammatory responses observed in various skin disorders. The introduction of senescent cells or skin cells from aged donors could also be developed to mimic ageing processes in the skin. Overall, we believe that this novel bioengineered model of human full thickness skin will be of value to the scientific community for basic research and screening applications.

## Supporting information


**Table S1.** Antibodies used for immunohistochemical staining. Samples were incubated with primary antibodies diluted in blocking buffer overnight at 4 °C. Samples were then incubated with the secondary antibodies diluted in blocking buffer for 1 h at room temperature. Antibodies were sourced from Abcam: www.abcam.com.
