# Supplementary material for: Bioengineering the microanatomy of human skin
Source: J Anat. 2019 Feb 10;234(4):438–55. doi: 10.1111/joa.12942 (PMC6422806; doi:10.1111/joa.12942)
Supplement: Supplementary file 1 — Table S1. Antibodies used for immunohistochemical staining. Samples were incubated with primary antibodies diluted in blocking buffer overnight at 4 °C. Samples were then incubated with the secondary antibodies diluted in blocking buffer for 1 h at room temperature. Antibodies were sourced from Abcam: www.abcam.com. [file JOA-234-438-s001.docx]

| Antibody | Supplier | Product Code | Dilution |
| --- | --- | --- | --- |
| Keratin 10 | Abcam | ab76318 | 1:100 |
| Keratin 14 | Abcam | ab7800 | 1:100 |
| Filaggrin | Abcam | ab17808 | 1:100 |
| Loricrin | Abcam | ab85679 | 1:100 |
| Involucrin | Abcam | ab53112 | 1:100 |
| SPRR1b | Abcam | ab123237 | 1:100 |
| Periplakin | Abcam | ab131269 | 1:100 |
| Claudin-1 | Abcam | ab15098 | 1:100 |
| Connexin 43 | Abcam | ab11370 | 1:100 |
| E-cadherin | Abcam | ab1416 | 1:100 |
| Collagen IV | Abcam | ab6586 | 1:100 |
| Collagen I | Abcam | ab34710 | 1:100 |
| Collagen III | Abcam | ab7778 | 1:100 |
| Ki67 | Abcam | ab16667 | 1:100 |
| p63 | Abcam | ab124762 | 1:100 |
| Integrin α6 | Abcam | ab181551 | 1:100 |
| Fibronectin | Abcam | ab23750 | 1:100 |
| Elastin | Abcam | ab9519 | 1:100 |
| Donkey Anti-Rabbit IgG Alexa Fluor^®^ 488 | Abcam | ab150073 | 1:1000 |
| Donkey Anti-Mouse IgG Alexa Fluor^®^ 488 | Abcam | ab150105 | 1:1000 |
| Donkey Anti-Rabbit IgG Alexa Fluor^®^ 594 | Abcam | ab150076 | 1:1000 |
| Donkey Anti-Mouse IgG Alexa Fluor^®^ 594 | Abcam | ab150108 | 1:1000 |

**Supplementary Table 1.**

Antibodies used for immunohistochemical staining. Samples were incubated with primary antibodies diluted in blocking buffer overnight at 4^o^C. Samples were then incubated with the secondary antibodies diluted in blocking buffer for 1 hour at room temperature. Antibodies were sourced from Abcam: www.abcam.com
